# Crystal structure of 3-acet­oxy-2-methyl­benzoic acid

**DOI:** 10.1107/S2056989015010865

**Published:** 2015-06-13

**Authors:** Matheswaran Saranya, Annamalai Subashini, Chidambaram Arunagiri, Packianathan Thomas Muthiah

**Affiliations:** a PG & Research Department of Chemistry, Seethalakshmi Ramaswami College, Tiruchirappalli 620 002, Tamil Nadu, India; b PG & Research Department of Physics, Government Arts College, Ariyalur 621 713, Tamil Nadu, India; c School of Chemistry, Bharathidasan University, Tiruchirappalli 620 024, Tamil Nadu, India

**Keywords:** crystal structure, ester, acet­oxy, benzoic acid, hydrogen bonding, graph-set motifs

## Abstract

In the title mol­ecule, C_10_H_10_O_4_, the carb­oxy­lic acid group is twisted by 11.37 (15)° from the plane of the benzene ring and the acet­oxy group is twisted from this plane by 86.60 (17)°. In the crystal, mol­ecules are linked by pairs of O—H⋯O hydrogen bonds, forming inversion dimers with the expected *R*_2_^2^(8) graph-set motif.

## Related literature   

For related structures, see: Chiari *et al.* (1981 ▸); Fronczek *et al.* (1982 ▸); Montis & Hursthouse (2012 ▸); Shoaib *et al.* (2014 ▸); Wheatley (1964 ▸).
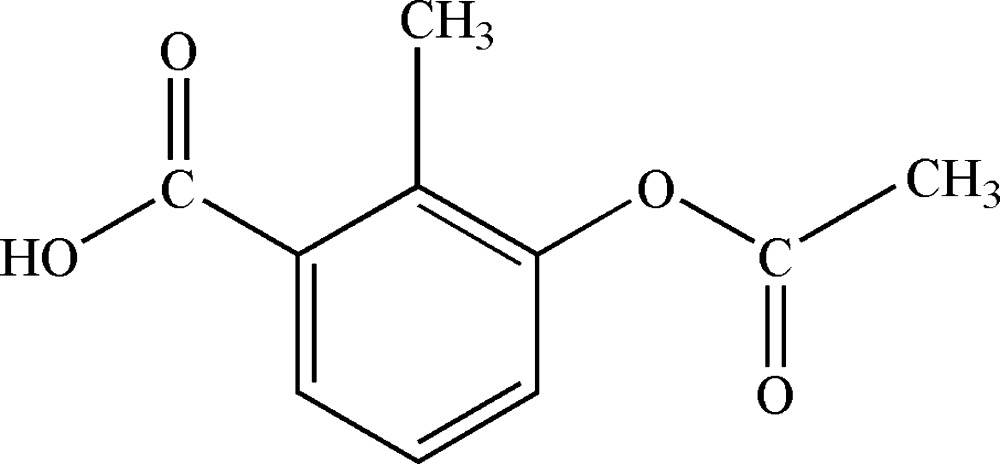


## Experimental   

### Crystal data  

C_10_H_10_O_4_
*M* *_r_* = 194.18
Orthorhombic, 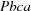

*a* = 7.754 (2) Å
*b* = 11.346 (3) Å
*c* = 21.187 (6) Å
*V* = 1864.0 (9) Å^3^
*Z* = 8
Mo *K*α radiation
μ = 0.11 mm^−1^
*T* = 293 K
0.38 × 0.22 × 0.06 mm

### Data collection  

Bruker SMART APEXII DUO CCD area-detector diffractometer
Absorption correction: multi-scan (*SADABS*; Bruker, 2009 ▸) *T*_min_ = 0.960, *T*_max_ = 0.994
14775 measured reflections
2131 independent reflections
1017 reflections with *I* > 2σ(*I*)
*R*_int_ = 0.095

### Refinement  

*R*[*F* ^2^ > 2σ(*F* ^2^)] = 0.063
*wR*(*F* ^2^) = 0.184
*S* = 1.03
2131 reflections
133 parameters
H atoms treated by a mixture of independent and constrained refinement
Δρ_max_ = 0.22 e Å^−3^
Δρ_min_ = −0.18 e Å^−3^

### 

Data collection: *APEX2* (Bruker, 2009 ▸); cell refinement: *SAINT* (Bruker, 2009 ▸); data reduction: *SAINT*; program(s) used to solve structure: *SHELXS97* (Sheldrick, 2008 ▸); program(s) used to refine structure: *SHELXL97* (Sheldrick, 2008 ▸); molecular graphics: *PLATON* (Spek, 2009 ▸); software used to prepare material for publication: *PLATON*.

## Supplementary Material

Crystal structure: contains datablock(s) global, I. DOI: 10.1107/S2056989015010865/lh5765sup1.cif

Structure factors: contains datablock(s) I. DOI: 10.1107/S2056989015010865/lh5765Isup2.hkl

Supporting information file. DOI: 10.1107/S2056989015010865/lh5765Isup3.cml

**Fig. 1.**
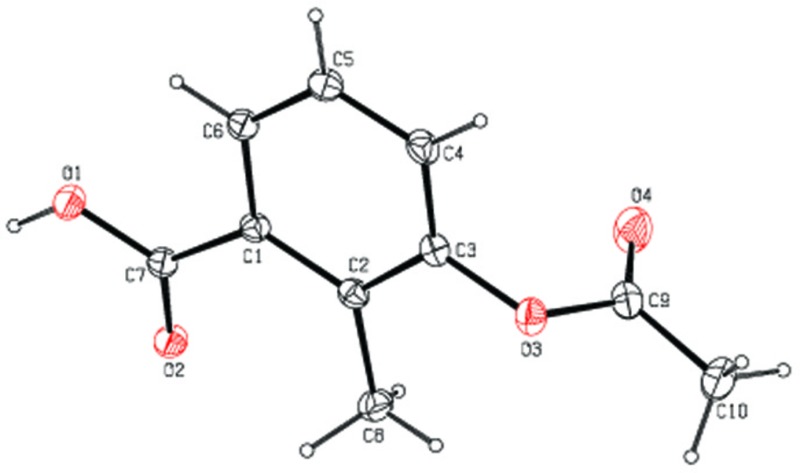
The molecular structure of the title compound with displacement ellipsoids drawn at the 30% probability level.

**Fig. 2.**
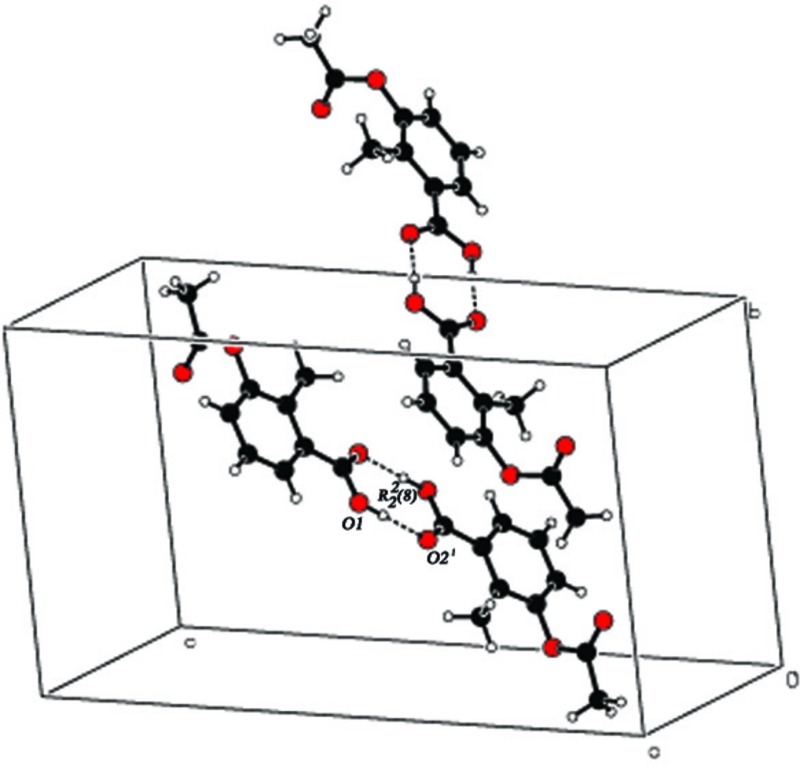
Part of the crystal structure with hydrogen bonds shown as dashed lines [symmetry code: (i) -x, -y + 2, -z + 1].

. DOI: 10.1107/S2056989015010865/lh5765fig1.tif
The mol­ecular structure of the title compound with displacement ellipsoids drawn at the 30% probability level.

x y z . DOI: 10.1107/S2056989015010865/lh5765fig2.tif
Part of the crystal structure with hydrogen bonds shown as dashed lines [symmetry code: (i) −*x*, −*y* + 2, −*z* + 1].

CCDC reference: 1405114

Additional supporting information:  crystallographic information; 3D view; checkCIF report

Enhanced figure: interactive version of Fig. 1

## Figures and Tables

**Table 1:** Hydrogen-bond geometry (, )

*D*H*A*	*D*H	H*A*	*D* *A*	*D*H*A*
O1H1O2^i^	0.93(5)	1.70(5)	2.622(3)	176(3)

Symmetry code: (i) 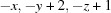.

## supplementary crystallographic information

### S1. Comment

Crystal structures of 2-acetoxy-3-methylbenzoic acid (3-methyl aspirin) (Chiari *et al.*, 1981) and 2-acetoxy-6-methylbenzoic acid (6-methyl aspirin) have already been reported in the literature (Fronczek *et al.* 1982). Aspirin is a unique drug as it is effective against pain, it has anti-pyretic and anti-inflammatory properties, and it is widely used during heart attacks or strokes. The crystal and molecular structure of aspirin has been reported by Wheatley in 1964. We report herein on the crystal structure of the title molecule.

The molecular structure of the title compound is shown in Fig. 1. There are some very definite angular distortions within the molecule, both in the benzene ring and the carboxyl group, but more particularly, in the acetyl group. The internal angle at C3 (123.6 (3)°) is greater than 120° (the expected value in terms of hybridization principles), and that at C2 is less (116.2 (2)°). The carboxyl group is bent away from the methyl and acetyl group, possibly by repulsion between O1 and O2, so that there is a substantial increase in the angle C2—C1—C7, and a decrease in C6—C1—C7. The angle O1—C7—O2 is greater than 120°, again suggesting repulsion between oxygen atoms. The carboxyl group is twisted by 11.37 (15)° out of the plane of the benzene ring, and the acetoxy group is twisted out of plane by 86.60 (17)°.

Certain torsion angles reveal conformational changes in the carboxyl and acetoxy groups caused by methyl substitution at C2. Atoms C7 and C8 lie in the plane of the benzene ring and O3 is slightly out of plane. The deviations of atoms C7, C8 and O3 from the least-squares plane of the benzene ring are -0.015 (3), 0.010 (3) and 0.111 (2) Å, respectively.

A similar situation is exists in 2-acetoxy-6-methyl benzoic acid (Fronczek *et al.* 1982). Comparison of the C2—C3—O3—C9 angle (94.8 (3)°) reveals that the acetoxy group is skewed slightly away from the methyl group in this structure. There is also a slight but significant twist in the ester backbone, C3—O3—C9—C10 = -178.3 (3)°, present in the title compound, a result quite similar to that in 6-methyl aspirin (Fronczek *et al.* 1982). In the crystal, pairs of O—H···O hydrogen bonds form inversion dimers with the expected R^2^_2_(8) graph-set motif (Fig. 2). The carboxyl oxygen atom O1 acts as a donor in an intermolecular hydrogen bond to atom O2, producing an *R*^2^_2_(8) ring, thus creating a hydrogen-bonded dimer. This type of motif is commonly observed (Shoaib *et al.*, 2014; Montis & Hursthouse *et al.*, 2012).

### S2. Experimental

A hot methanol solution (20 ml) of 3-acetoxy-2-methyl benzoic acid [3 A2MBA] (1 mm 0.194 g, Alfa aesar) was stirred at room temperature for 20 minutes. The resulting solution was kept as such for crystallization. After a few days colourless block-shaped crystals were appeared from the mother liquor.

### S3. Refinement

H atoms bonded to C atoms were positioned geometrically and treated as riding with C—H = 0.93–0.96Å and U_iso_(H) = 1.2U_eq_(C) or 1.5U_eq_(C_methyl_). The hydroxyl H atom was refined independently with an isotropic displacement parameter.

### Crystal data

**Table:**

C_10_H_10_O_4_	*F*(000) = 816
*M**_r_* = 194.18	*D*_x_ = 1.384 Mg m^−^^3^
Orthorhombic, *P**b**c**a*	Mo *K*α radiation, λ = 0.71073 Å
Hall symbol: -P 2ac 2ab	θ = 1.9–27.5°
*a* = 7.754 (2) Å	µ = 0.11 mm^−^^1^
*b* = 11.346 (3) Å	*T* = 293 K
*c* = 21.187 (6) Å	Block, colourless
*V* = 1864.0 (9) Å^3^	0.38 × 0.22 × 0.06 mm
*Z* = 8	

### Data collection

**Table:**

Bruker SMART APEXII DUO CCD area-detector diffractometer	2131 independent reflections
Radiation source: fine-focus sealed tube	1017 reflections with *I* > 2σ(*I*)
Graphite monochromator	*R*_int_ = 0.095
φ and ω scans	θ_max_ = 27.5°, θ_min_ = 1.9°
Absorption correction: multi-scan (*SADABS*; Bruker, 2009)	*h* = −10→10
*T*_min_ = 0.960, *T*_max_ = 0.994	*k* = −13→14
14775 measured reflections	*l* = −27→27

### Refinement

**Table:**

Refinement on *F*^2^	Primary atom site location: structure-invariant direct methods
Least-squares matrix: full	Secondary atom site location: difference Fourier map
*R*[*F*^2^ > 2σ(*F*^2^)] = 0.063	Hydrogen site location: inferred from neighbouring sites
*wR*(*F*^2^) = 0.184	H atoms treated by a mixture of independent and constrained refinement
*S* = 1.03	*w* = 1/[σ^2^(*F*_o_^2^) + (0.0778*P*)^2^ + 0.1948*P*] where *P* = (*F*_o_^2^ + 2*F*_c_^2^)/3
2131 reflections	(Δ/σ)_max_ < 0.001
133 parameters	Δρ_max_ = 0.22 e Å^−^^3^
0 restraints	Δρ_min_ = −0.18 e Å^−^^3^

### Special details

**Table:**

Geometry. Bond distances, angles *etc*. have been calculated using the rounded fractional coordinates. All su's are estimated from the variances of the (full) variance-covariance matrix. The cell e.s.d.'s are taken into account in the estimation of distances, angles and torsion angles
Refinement. Refinement on *F*^2^ for ALL reflections except those flagged by the user for potential systematic errors. Weighted *R*-factors *wR* and all goodnesses of fit *S* are based on *F*^2^, conventional *R*-factors *R* are based on *F*, with *F* set to zero for negative *F*^2^. The observed criterion of *F*^2^ > σ(*F*^2^) is used only for calculating -*R*-factor-obs *etc*. and is not relevant to the choice of reflections for refinement. *R*-factors based on *F*^2^ are statistically about twice as large as those based on *F*, and *R*-factors based on ALL data will be even larger.

### Fractional atomic coordinates and isotropic or equivalent isotropic displacement parameters (Å^2^)

**Table:**

	*x*	*y*	*z*	*U*_iso_*/*U*_eq_	
O1	0.2256 (3)	0.9740 (2)	0.49706 (10)	0.0671 (8)	
O2	0.0048 (2)	0.88776 (19)	0.45072 (9)	0.0665 (8)	
O3	0.3342 (3)	0.54490 (19)	0.34035 (9)	0.0677 (8)	
O4	0.3190 (4)	0.6303 (3)	0.24740 (11)	0.1057 (13)	
C1	0.2878 (3)	0.8175 (3)	0.42969 (12)	0.0500 (9)	
C2	0.2399 (3)	0.7163 (3)	0.39650 (12)	0.0523 (10)	
C3	0.3732 (4)	0.6518 (3)	0.36983 (12)	0.0547 (10)	
C4	0.5439 (4)	0.6833 (3)	0.37411 (13)	0.0659 (13)	
C5	0.5868 (4)	0.7823 (3)	0.40696 (14)	0.0658 (11)	
C6	0.4601 (3)	0.8482 (3)	0.43437 (13)	0.0565 (10)	
C7	0.1608 (4)	0.8942 (3)	0.45996 (12)	0.0534 (10)	
C8	0.0586 (4)	0.6754 (3)	0.38841 (14)	0.0688 (11)	
C9	0.3081 (4)	0.5436 (4)	0.27810 (16)	0.0690 (14)	
C10	0.2636 (5)	0.4254 (3)	0.25496 (17)	0.0927 (17)	
H1	0.141 (6)	1.022 (4)	0.514 (2)	0.139 (18)*	
H4	0.62880	0.63780	0.35490	0.0790*	
H5	0.70160	0.80480	0.41070	0.0790*	
H6	0.49030	0.91550	0.45680	0.0680*	
H8A	0.00260	0.72250	0.35680	0.1030*	
H8B	−0.00190	0.68300	0.42770	0.1030*	
H8C	0.05830	0.59430	0.37540	0.1030*	
H10A	0.23420	0.42970	0.21100	0.1390*	
H10B	0.16700	0.39560	0.27840	0.1390*	
H10C	0.36050	0.37380	0.26040	0.1390*	

### Atomic displacement parameters (Å^2^)

**Table:**

	*U*^11^	*U*^22^	*U*^33^	*U*^12^	*U*^13^	*U*^23^
O1	0.0434 (12)	0.0748 (16)	0.0830 (14)	0.0033 (11)	−0.0008 (10)	−0.0255 (12)
O2	0.0386 (12)	0.0773 (15)	0.0836 (14)	0.0004 (10)	0.0012 (9)	−0.0192 (12)
O3	0.0830 (15)	0.0599 (15)	0.0602 (13)	0.0060 (12)	0.0015 (10)	−0.0054 (10)
O4	0.151 (3)	0.102 (2)	0.0642 (15)	−0.014 (2)	−0.0150 (15)	0.0037 (16)
C1	0.0414 (15)	0.0559 (18)	0.0527 (15)	0.0009 (13)	0.0003 (11)	−0.0004 (13)
C2	0.0460 (16)	0.0602 (19)	0.0507 (15)	0.0017 (14)	0.0011 (12)	0.0033 (14)
C3	0.0574 (19)	0.0575 (19)	0.0492 (15)	0.0071 (15)	0.0023 (12)	−0.0001 (14)
C4	0.0500 (19)	0.082 (3)	0.0658 (19)	0.0084 (17)	0.0086 (13)	−0.0084 (17)
C5	0.0405 (16)	0.079 (2)	0.078 (2)	0.0027 (16)	0.0030 (14)	−0.0089 (18)
C6	0.0455 (16)	0.0625 (19)	0.0615 (17)	0.0021 (14)	0.0026 (13)	−0.0065 (15)
C7	0.0439 (17)	0.0598 (19)	0.0565 (16)	0.0001 (15)	0.0012 (13)	−0.0051 (14)
C8	0.0564 (19)	0.070 (2)	0.080 (2)	−0.0075 (16)	0.0007 (14)	−0.0101 (17)
C9	0.069 (2)	0.079 (3)	0.059 (2)	0.0072 (19)	0.0002 (15)	−0.0072 (19)
C10	0.095 (3)	0.090 (3)	0.093 (3)	−0.001 (2)	−0.010 (2)	−0.030 (2)

### Geometric parameters (Å, º)

**Table:**

O1—C7	1.300 (4)	C4—C5	1.363 (5)
O2—C7	1.228 (3)	C5—C6	1.364 (4)
O3—C3	1.397 (4)	C9—C10	1.469 (6)
O3—C9	1.334 (4)	C4—H4	0.9300
O4—C9	1.182 (5)	C5—H5	0.9300
O1—H1	0.93 (5)	C6—H6	0.9300
C1—C2	1.397 (4)	C8—H8A	0.9600
C1—C6	1.384 (3)	C8—H8B	0.9600
C1—C7	1.462 (4)	C8—H8C	0.9600
C2—C8	1.490 (4)	C10—H10A	0.9600
C2—C3	1.387 (4)	C10—H10B	0.9600
C3—C4	1.374 (4)	C10—H10C	0.9600
			
O1···O2^i^	2.622 (3)	C7···C5^ix^	3.506 (4)
O2···C7^i^	3.369 (4)	C7···O3^ii^	3.057 (4)
O2···C8	2.779 (4)	C8···C9	3.383 (5)
O2···O1^i^	2.622 (3)	C8···O2	2.779 (4)
O2···O3^ii^	3.195 (3)	C9···C8	3.383 (5)
O3···C7^iii^	3.057 (4)	C10···O4^iii^	3.413 (5)
O3···O2^iii^	3.195 (3)	C6···H8C^ii^	3.0600
O3···C1^iii^	3.337 (4)	C6···H8B^viii^	2.9600
O4···C4^iv^	3.397 (4)	C7···H8B	2.7900
O4···C4	3.257 (4)	C7···H1^i^	2.59 (5)
O4···C10^ii^	3.413 (5)	C9···H8C	2.8900
O4···C2	3.363 (4)	H1···O1^i^	2.85 (5)
O1···H6	2.3200	H1···O2^i^	1.70 (5)
O1···H6^v^	2.7200	H1···C7^i^	2.59 (5)
O1···H1^i^	2.85 (5)	H1···H1^i^	2.32 (7)
O2···H5^vi^	2.6700	H4···O4^vii^	2.6200
O2···H8B	2.3700	H5···O2^x^	2.6700
O2···H1^i^	1.70 (5)	H6···O1	2.3200
O2···H8A	2.7300	H6···O1^v^	2.7200
O3···H8C	2.3300	H8A···O2	2.7300
O4···H4^iv^	2.6200	H8A···O4^iv^	2.8300
O4···H8A^vii^	2.8300	H8B···O2	2.3700
C1···O3^ii^	3.337 (4)	H8B···C7	2.7900
C2···O4	3.363 (4)	H8B···C6^ix^	2.9600
C3···C7^iii^	3.501 (5)	H8C···O3	2.3300
C4···O4	3.257 (4)	H8C···C9	2.8900
C4···O4^vii^	3.397 (4)	H8C···C6^iii^	3.0600
C5···C7^viii^	3.506 (4)	H10B···H10C^iv^	2.5300
C7···C3^ii^	3.501 (5)	H10C···H10B^vii^	2.5300
C7···O2^i^	3.369 (4)		
			
C3—O3—C9	119.0 (3)	O3—C9—O4	121.6 (4)
C7—O1—H1	112 (3)	C3—C4—H4	121.00
C2—C1—C7	122.1 (2)	C5—C4—H4	121.00
C6—C1—C7	118.0 (3)	C4—C5—H5	120.00
C2—C1—C6	120.0 (3)	C6—C5—H5	120.00
C1—C2—C8	124.4 (2)	C1—C6—H6	119.00
C3—C2—C8	119.5 (3)	C5—C6—H6	119.00
C1—C2—C3	116.2 (2)	C2—C8—H8A	109.00
O3—C3—C2	118.6 (3)	C2—C8—H8B	109.00
O3—C3—C4	117.6 (3)	C2—C8—H8C	109.00
C2—C3—C4	123.6 (3)	H8A—C8—H8B	110.00
C3—C4—C5	118.9 (3)	H8A—C8—H8C	109.00
C4—C5—C6	119.6 (3)	H8B—C8—H8C	110.00
C1—C6—C5	121.8 (3)	C9—C10—H10A	109.00
O1—C7—C1	114.8 (3)	C9—C10—H10B	109.00
O2—C7—C1	123.9 (3)	C9—C10—H10C	109.00
O1—C7—O2	121.3 (3)	H10A—C10—H10B	110.00
O3—C9—C10	112.1 (3)	H10A—C10—H10C	109.00
O4—C9—C10	126.4 (3)	H10B—C10—H10C	110.00
			
C9—O3—C3—C2	94.8 (3)	C2—C1—C7—O2	−12.0 (5)
C9—O3—C3—C4	−89.2 (3)	C6—C1—C7—O1	−10.6 (4)
C3—O3—C9—O4	0.8 (5)	C6—C1—C7—O2	167.6 (3)
C3—O3—C9—C10	−178.3 (3)	C1—C2—C3—O3	175.0 (2)
C6—C1—C2—C3	0.1 (4)	C1—C2—C3—C4	−0.7 (4)
C6—C1—C2—C8	−179.8 (3)	C8—C2—C3—O3	−5.1 (4)
C7—C1—C2—C3	179.7 (3)	C8—C2—C3—C4	179.2 (3)
C7—C1—C2—C8	−0.2 (4)	O3—C3—C4—C5	−174.9 (3)
C2—C1—C6—C5	0.4 (4)	C2—C3—C4—C5	0.9 (5)
C7—C1—C6—C5	−179.3 (3)	C3—C4—C5—C6	−0.5 (5)
C2—C1—C7—O1	169.8 (3)	C4—C5—C6—C1	−0.2 (5)

Symmetry codes: (i) −*x*, −*y*+2, −*z*+1; (ii) −*x*+1/2, *y*+1/2, *z*; (iii) −*x*+1/2, *y*−1/2, *z*; (iv) *x*−1/2, *y*, −*z*+1/2; (v) −*x*+1, −*y*+2, −*z*+1; (vi) *x*−1, *y*, *z*; (vii) *x*+1/2, *y*, −*z*+1/2; (viii) *x*+1/2, −*y*+3/2, −*z*+1; (ix) *x*−1/2, −*y*+3/2, −*z*+1; (x) *x*+1, *y*, *z*.

### Hydrogen-bond geometry (Å, º)

**Table:**

*D*—H···*A*	*D*—H	H···*A*	*D*···*A*	*D*—H···*A*
O1—H1···O2^i^	0.93 (5)	1.70 (5)	2.622 (3)	176 (3)

Symmetry code: (i) −*x*, −*y*+2, −*z*+1.
