# Supplementary figures and images for: Crystal structure of 3-acet­oxy-2-methyl­benzoic acid
Source: Acta Crystallogr E Crystallogr Commun. 2015 Jun 13;71(Pt 7):o474. doi: 10.1107/S2056989015010865 (PMC4518910; doi:10.1107/S2056989015010865)

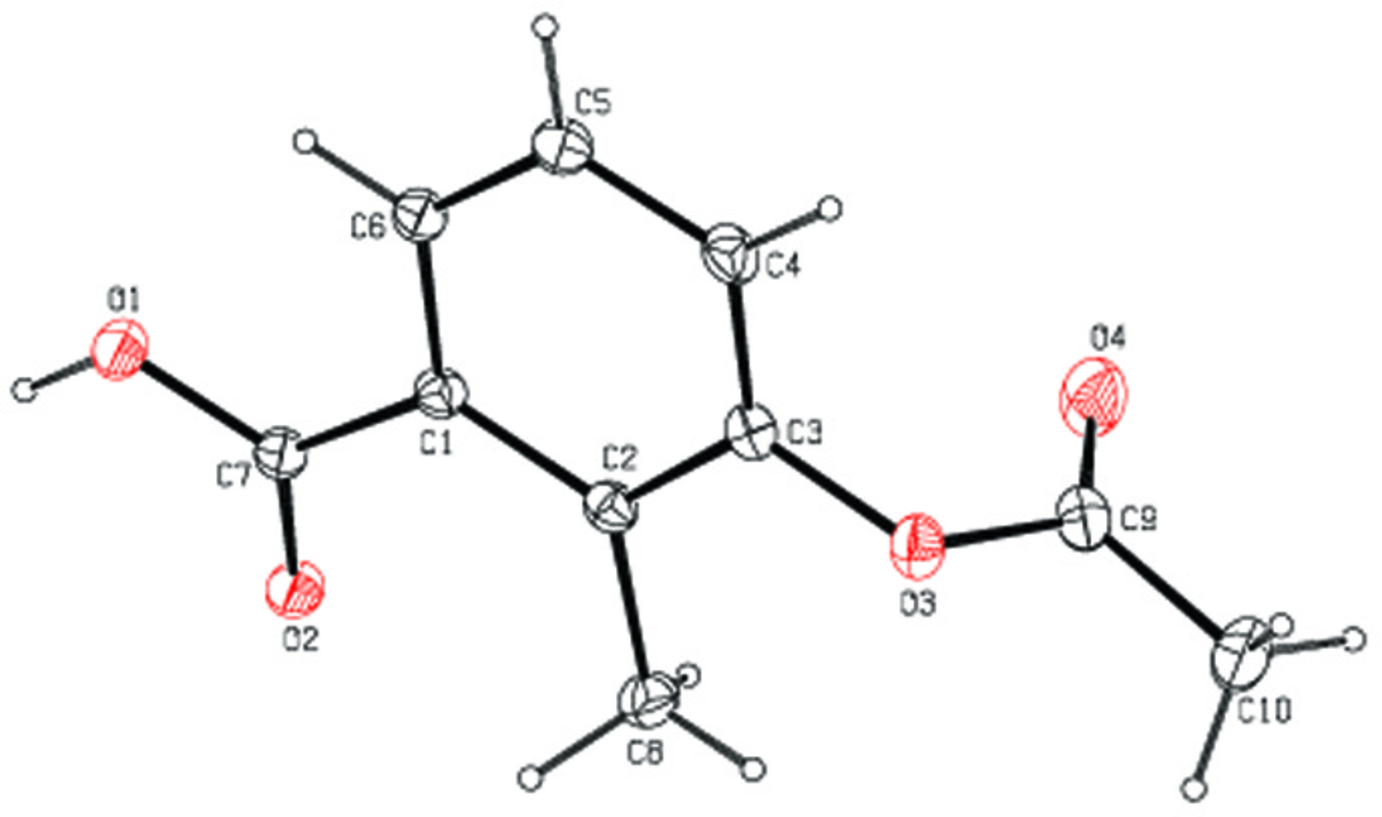

Supplement: Supplementary file 4 [file e-71-0o474-fig1.tif]

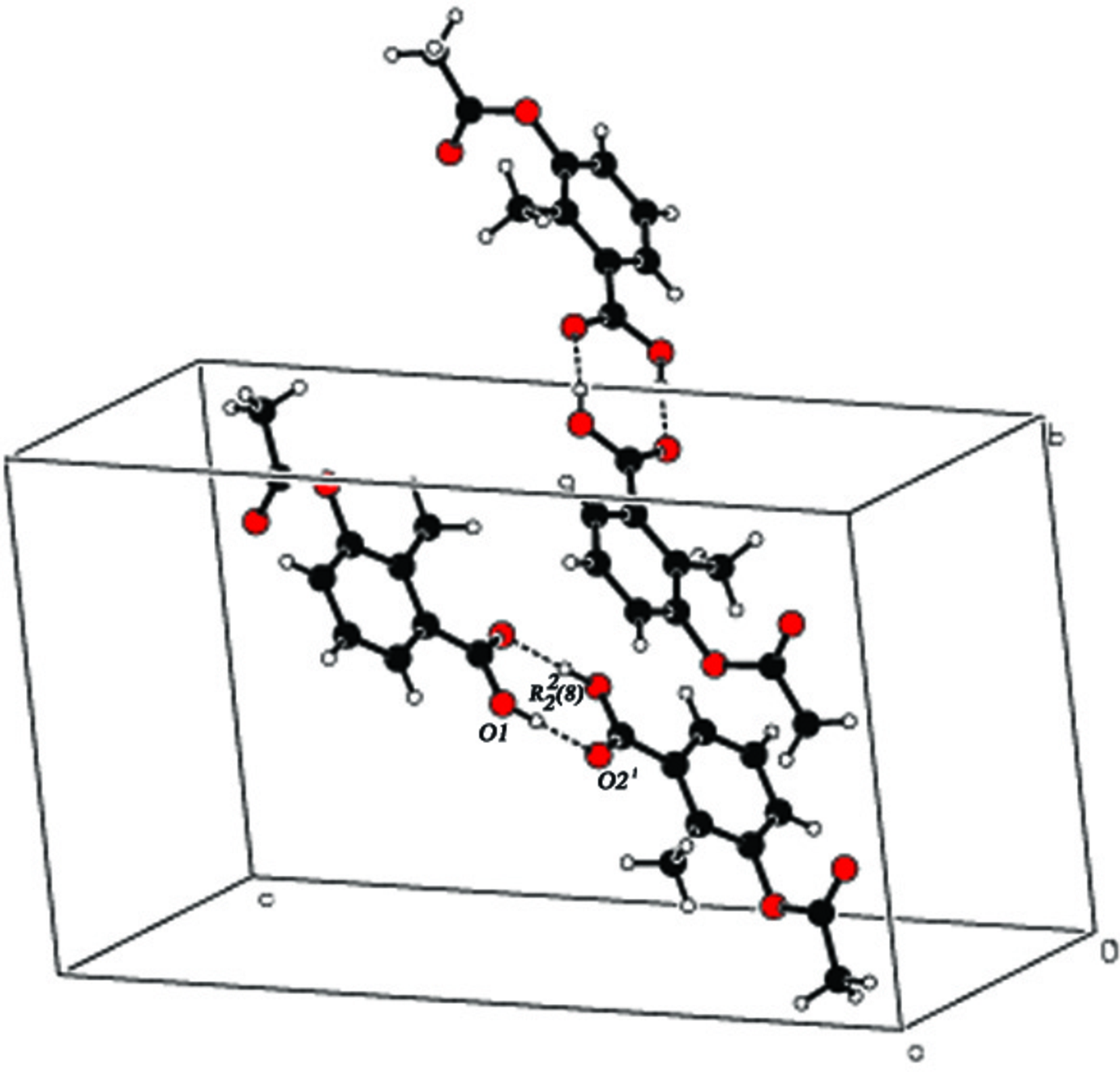

Supplement: Supplementary file 5 [file e-71-0o474-fig2.tif]
